# The U. S. Pharmacopœia

**Published:** 1873-04

**Authors:** 


*The Pharmacopœia of the United States. Fifth Decennial Edition.* Philadelphia: J. B. Lippincott & Co.. 1873. Buffalo: H. H. Otis.

The Pharmacopœia as it comes to us in its fifth decennial revision is an improvement over the former edition. Several articles have been dropped and a few new ones added. The nomenclature has been changed, and other manifest improvements have been made in the general construction of the work.

The work of revising the Pharmacopœia in such a manner as to suit all, would be undoubtedly an impossible task, yet we think that a few of the drugs whose names are still incorporated within its limits, might have been left out without any serious detriment to the value of the work. As it is however, we are happy to accept the work in its new and improved form, and are willing to have time work out the changes neglected by the convention for its revision.

